# Global Research Trends of Gender-Related Artificial Intelligence in Medicine Between 2001–2020: A Bibliometric Study

**DOI:** 10.3389/fmed.2022.868040

**Published:** 2022-05-17

**Authors:** Ha Young Yoon, Heisook Lee, Jeong Yee, Hye Sun Gwak

**Affiliations:** ^1^College of Pharmacy and Graduate School of Pharmaceutical Sciences, Ewha Womans University, Seoul, South Korea; ^2^Korea Center for Gendered Innovations for Science and Technology Research, Seoul, South Korea

**Keywords:** artificial intelligence, bibliometric analysis, gender, medicine, medical research

## Abstract

This study aimed to assess the research on medical Artificial intelligence (AI) related to sex/gender and explore global research trends over the past 20 years. We searched the Web of Science (WoS) for gender-related medical AI publications from 2001 to 2020. We extracted the bibliometric data and calculated the annual growth of publications, Specialization Index, and Category Normalized Citation Impact. We also analyzed the publication distributions by institution, author, WoS subject category, and journal. A total of 3,110 papers were included in the bibliometric analysis. The number of publications continuously increased over time, with a steep increase between 2016 and 2020. The United States of America and Harvard University were the country and institution that had the largest number of publications. Surgery and urology nephrology were the most common subject categories of WoS. The most occurred keywords were machine learning, classification, risk, outcomes, diagnosis, and surgery. Despite increased interest, gender-related research is still low in medical AI field and further research is needed.

## Introduction

Gender medicine investigates the influence of sex/gender on the pathophysiology, prevention and treatment of disease, and the social and psychological aspects of illness (1, 2). Although medical research has been performed dominantly on men both in preclinical and clinical studies (3), there have been continuous efforts to overcome this gender bias (4). Since Healy B proposed gender differences in clinical outcomes (5), the subject has been discussed extensively, including in medical fields such as cardiovascular and gastrointestinal disease and oncology (6–9).

Artificial intelligence (AI) is a branch of computer science in which machines are developed to mimic human intelligence, including cognition, perception, and problem-solving (10, 11). This field has developed quickly and been applied to many areas, including medicine (11, 12). With its sophisticated algorithms, AI assists doctors and health professionals with data management, image-based diagnostics, robotic surgery, prediction models, and decision-making support (13, 14).

The widespread application of AI has promoted research in related fields, supporting the implementation of AI technologies in health care (14). Guo et al. found that publications on health care related to AI increased an average of 17% per year since 1995, with a steep increase of 45% between 2014 and 2019 (15). Along with the increased number of publications in medical AI, gender differences are important in other research areas. As bibliometric analysis quantitatively analyzes scientific publications, it can provide researchers and stakeholders with a macroscopic overview of research trends and help develop further research direction and policy. This study aims to assess the research activity on medical AI related to sex/gender and explore the global research trends over the past 20 years.

## Methods

We extracted bibliographic data on gender-related medical AI articles from Web of Science (WoS) Core Collection. WoS Core Collection, which contains over 20,000 peer-reviewed, high-quality journals published worldwide covering various fields (16), is one of the most well-established and commonly used databases for bibliometric analysis (17, 18). Articles from 2001 to 2020 were collected using the following search terms: {TS=(“artificial intelligence” OR “machine intelligence” OR “artificial neural network^*^” OR “machine learning” OR “deep learn^*^” OR “natural language process^*^” OR “robotic^*^” OR “thinking computer system” OR “fuzzy expert system^*^” OR “evolutionary computation” OR “hybrid intelligent system^*^”)} AND {TS=(disease^*^ OR illness OR health-related OR medic^*^ OR “medical diagnosis” OR treatment OR health^*^ OR wellness OR well-being OR prescription OR drug)} AND {TS=(gender OR sex OR male OR female)}.

The inclusion criteria were: (i) articles, review articles, and editorial materials; (ii) publications from 2001 to 2020; and (iii) full texts published in English. Articles were excluded if they were a proceeding paper, meeting abstract, book review, book chapter, or correction.

For bibliometric analysis, we extracted the title, abstract, year of publication, journal name with impact factor, authors, institution, country, WoS subject category, keywords, and number of citations. We determined the annual publication growth, the relative research interest (RRI), and percentage of gender-related articles in the medical AI area. Four 5-year periods (2001–2005, 2006–2010, 2011–2015, and 2016–2020) were used to compare the progress of each country. Two bibliometric indicators, Specialization Index (SI) and Category Normalized Citation Impact (CNCI), were computed by InCites with the following equation (19, 20):


$$\begin{array}{lll} SI & = & \frac{Share\;\left(\%\right)\;of\;publications\;of\;region\;X}{Share\;\left(\%\right)\;of\;world\;publications\;in\;the\;same\;field}\\ CNCI & = & \;\frac{Observed\;citation\;rate\;of\;region\;X}{Expected\;citation\;rate\;in\;the\;same\;field,\;year,\;and\;documentation\;type} \end{array}$$

We also analyzed the publication distributions by institution, author, WoS subject category, and journal. We used VOSviewer (Leiden University, Leiden, The Netherlands; version 1.6.11) to draw network visualization maps and performed a citation analysis to identify the most cited articles.

## Results

### Publication Growth

**Figure 1:**
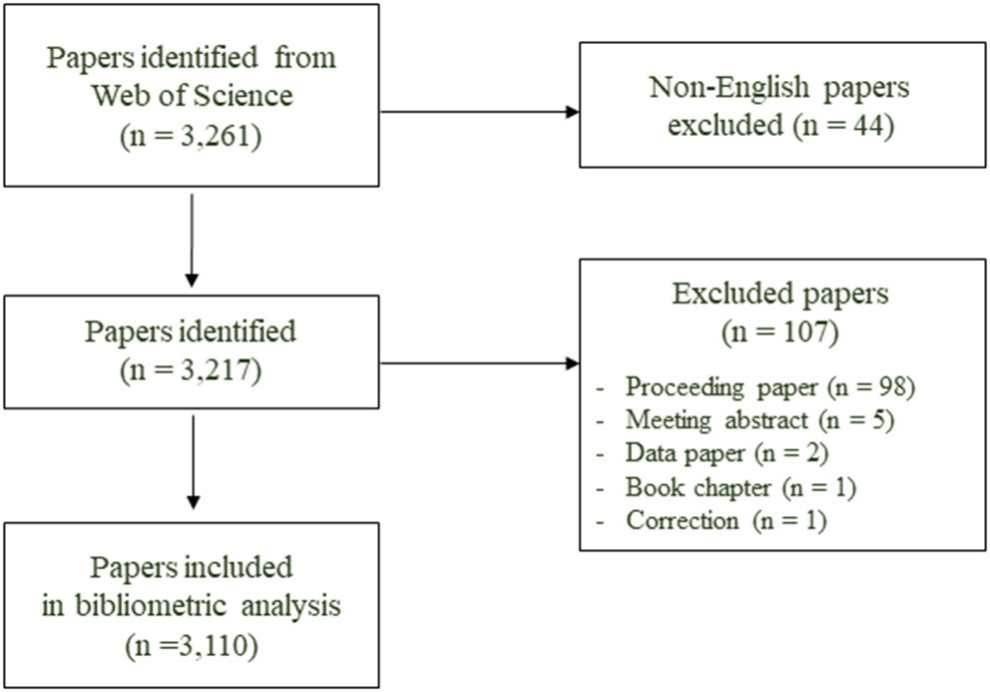
Selection process.

**Figure 2:**
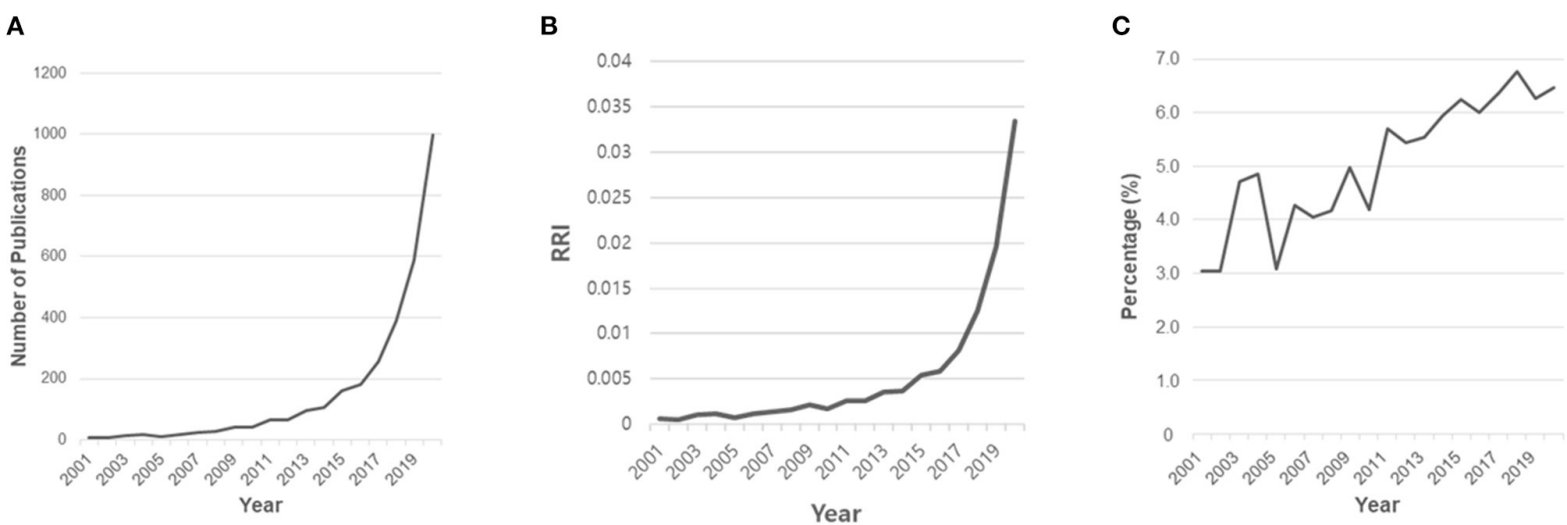
Annual growth of gender-related publications in medical AI. **(A)** The number of publications by year. **(B)** Relative research interest by year. **(C)** The percentage of gender-related publications in medical AI.

We identified 3,261 papers during the search (Figure 1). After excluding 44 non-English papers and 107 non-articles, 3,110 papers met the inclusion criteria. The graphs of absolute number of publications (Figure 2A) and RRI (Figure 2B) showed that the overall trend of publication increased from 2001 to 2020. The growth rates from 2001 to 2005, from 2006 to 2010, from 2011 to 2015, and from 2016 to 2020 were 71.4, 115.8, 146.2, and 453.3%, respectively. The number of publications increased steeply between 2016 and 2020, accounting for 77.5% (2,410/3,110) of all included papers. Figure 2C shows the percentages of gender-related articles in medical AI researches, which doubled to 6.5% from 2001 to 2020. The linear regression analysis showed that the percentages increased significantly over the last 20 years (*t* = 12.978, *P* < 0.001).

### Distribution by Country

Table 1 lists the top 20 countries which published gender-related articles in medical AI between 2001 and 2020. The United States of America (USA) had the most publications on gender-related medical AI (*n* = 1,377; 44.3%), followed by People's Republic of China (Peoples R China, *n* = 305; 9.8%), United Kingdom (*n* = 241; 7.7%), Italy (*n* = 211; 6.8%), and the Republic of Korea (South Korea, *n* = 201; 6.5%). Across the four five-year periods from 2001 to 2020, there was a 43.6% increase in the number of publications worldwide from the first to the last period. Canada had the greatest percentage increase in the number of publications (+134.0%), followed by Peoples R China (+127.5%), the South Korea (+77.5%), and United Kingdom (+65.0%). There was no country where the number of publications decreased.

**Table 1:** The 20 countries contributing the most gender-related articles in medical artificial intelligence.

**Country**	**Total number of papers (%)**	**2001–2005**			**2006–2010**			**2011–2015**			**2016–2020**			**Change between first and fourth 5-year periods (%)**
		***N***	**SI**	**CNCI**	***N***	**SI**	**CNCI**	***N***	**SI**	**CNCI**	***N***	**SI**	**CNCI**	
World		54		1.10	153		1.39	493		1.48	2,410		1.49	43.6
USA	1,377 (44.3)	19	1.02	1.26	78	1.78	1.87	220	1.41	1.86	1,059	1.81	1.79	54.7
Peoples R China	305 (9.8)	2	0.79	1.74	8	0.62	0.79	25	0.33	0.87	257	0.66	1.48	127.5
UK	241 (7.7)	3	0.74	1.55	10	0.95	1.51	30	0.75	2.24	198	1.28	2.64	65.0
Italy	211 (6.8)	8	3.85	0.47	15	2.53	2.11	33	1.41	0.89	155	1.63	1.87	18.4
South Korea	201 (6.5)	2	1.81	1.08	3	0.78	2.42	39	2.36	1.67	157	2.42	1.67	77.5
Germany	200 (6.4)	6	1.53	0.70	8	0.80	1.19	26	0.71	1.35	160	1.16	2.64	25.7
Canada	164 (5.3)	1	0.43	2.04	5	0.78	1.25	23	0.97	1.24	135	1.48	1.66	134.0
Turkey	121 (3.9)	2	3.64	4.31	9	4.14	0.35	21	2.14	0.43	89	2.20	0.96	43.5
Netherlands	106 (3.4)	2	1.67	0.28	2	0.60	1.14	8	0.61	2.69	94	1.86	3.67	46.0
India	101 (3.2)	2	1.96	0.58	2	0.52	1.86	9	0.40	0.90	88	0.84	1.20	43.0
Australia	94 (3.0)	–	–	–	1	0.23	0.19	13	0.64	3.15	80	0.94	2.02	–
France	91 (2.9)	2	0.75	2.01	10	1.45	1.13	16	0.64	0.86	63	0.70	2.05	30.5
Japan	88 (2.8)	3	0.74	1.19	5	0.58	0.84	16	0.59	1.00	64	0.65	0.94	20.3
Spain	85 (2.7)	2	1.37	0.48	3	0.60	1.57	11	0.53	0.90	69	0.84	1.44	33.5
Taiwan	74 (2.4)	3	4.31	0.17	4	1.69	0.60	10	1.15	1.23	57	2.01	1.35	18.0
Brazil	65 (2.1)	1	1.27	0.31	–	–	–	12	0.85	1.87	52	0.84	1.52	51.0
Iran	62 (2.0)	2	11.79	0.81	3	2.27	0.52	10	1.17	1.64	47	1.10	0.95	22.5
Switzerland	62 (2.0)	–	–	–	2	0.85	1.46	7	0.72	1.51	53	1.34	2.29	–
Sweden	56 (1.8)	–	–	–	1	0.49	3.38	11	1.32	2.34	44	1.30	2.07	–
Belgium	52 (1.7)	–	–	–	6	3.31	1.74	9	1.24	1.51	37	1.31	4.11	–

*CNCI, Category Normalized Citation Impact; N, number; SI, Specialization Index; Peoples R China, People's Republic of China; UK, United Kingdom; USA, United States of America*.

The SIs and CNCIs varied across countries and over time. The global CNCI increased steadily from 1.1 to 1.49 over the last 20 years. Compared to the first period (2001–2005), the USA, United Kingdom, South Korea, and Netherland showed an increase in both SI and CNCI in the fourth period (2016–2020), whereas Peoples R China, Turkey, and Japan showed a decrease in their SIs and CNCIs. From 2016 through 2020, South Korea had the highest SI (2.42), whereas Belgium had the highest CNCI (4.11).

### Distribution by Institution

Table 2 shows the top 10 institutions for gender-related articles in medical AI fields. The top 10 institutions contributed to 26.5% (824/3,110) of the total number of publications. Harvard University had the largest number of publications (*n* = 142; 4.6%), followed by the University of California System (*n* = 136; 4.4%), the University of Texas System (*n* = 84; 2.7%), Harvard Medical School (*n* = 84; 2.7%), and University of London (*n* = 81; 2.6%). Almost 90% of the top 10 institutions were located in the USA.

**Table 2:** The institutions contributing the most gender-related articles in medical artificial intelligence.

**Rank**	**Institution**	**Frequency**	**%**	**Country**
1	Harvard University	142	4.6	USA
2	University of California System	136	4.4	USA
3	University of Texas System	84	2.7	USA
3	Harvard Medical School	84	2.7	USA
5	University of London	81	2.6	UK
6	US Department of Veterans Affairs	65	2.1	USA
6	Pennsylvania Commonwealth System of Higher Education Pcshe	64	2.1	USA
8	Veterans Health Administration	59	1.9	USA
9	Stanford University	55	1.8	USA
10	Cleveland Clinic Foundation	54	1.7	USA

*UK, United Kingdom; USA, United States of America*.

**Figure 3:**
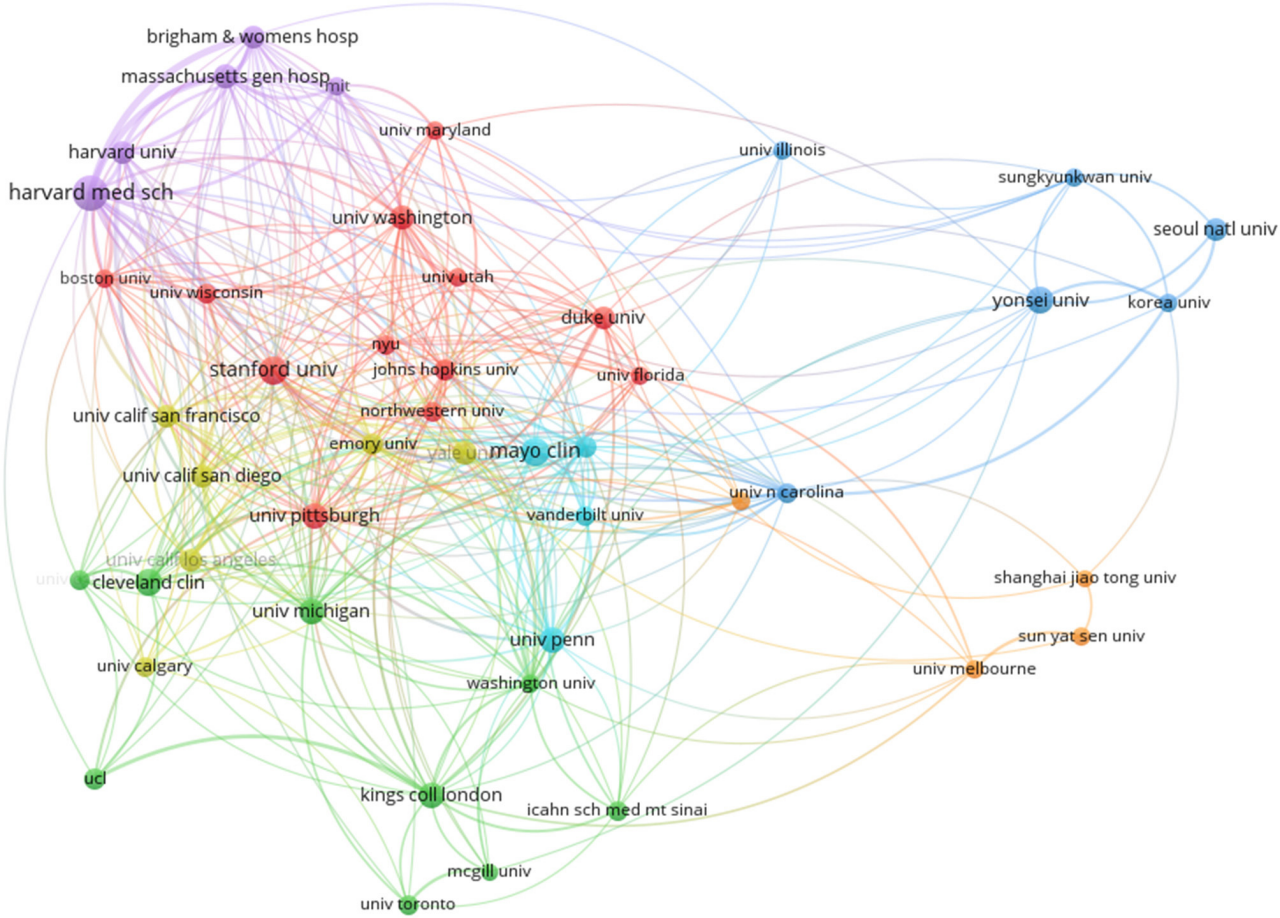
Network visualization map of the institutions.

Figure 3 shows the collaboration network between institutions. The network map of institutions that had at least 20 publications showed seven clusters. Among these, the four biggest clusters were (i) the cluster (red) on Stanford University and University of Pittsburgh; (ii) the cluster (green) on the Cleveland Clinic and the University of Michigan; (iii) the cluster (blue) on Yonsei University and Seoul National University; and (iv) the cluster (yellowish-green) on Yale University and the University of California (UC) San Diego.

### Distribution by Author

A total of 18,247 authors accounted for all publications for gender-related medical AI in 2001–2020. Dey D, Kaouk JH, and Grossie E contributed the most, with 10 publications, followed by Slomka PJ and Kaouk J, with nine publications (Table 3). In terms of first-author publications, Lin E ranked first with five publications, whereas Lee BJ ranked second with four. Most of the high-ranked authors by publications were from the USA, except for two from Europe. For the high-ranked first-authors, six were from Asia, four from the USA, and two from Europe. In addition, the results of co-citation, bibliometric coupling, and co-authorship analysis were shown in Supplementary Figure 1.

**Table 3:** The authors and first-authors contributing the most gender-related articles in medical artificial intelligence.

**Rank**	**Authors**	**Number of papers**	**Affiliation**	**Country**
**High-ranked authors**
1	Dey, Damini	10	Cedars-Sinai Medical Center	USA
1	Kaouk, Jihad H.	10	Glickman Urological Institute	USA
1	Grossi, Enzo	10	Semeion Center	Italy
4	Slomka, Piotr J.	9	Department of Imaging and Medicine and the Smidt Heart Institute	USA
4	Kaouk, Jihad	9	Cleveland Clin, Glickman Urol and Kidney Inst	USA
6	Berman, Daniel S.	8	Smidt Heart Institute and Biomedical Imaging Research Institute	USA
6	Stewart, Robert	8	South London and Maudsley NHS Foundation Trust	UK
8	Schoepf, U. Joseph	7	Medical University of South Carolina	USA
8	Garisto, Juan	7	Glickman Urological and Kidney Institute	USA
**High-ranked first authors**
1	Lin, Eugene	5	Vita Genomics Incorporated	Taiwan
2	Lee, Bum Ju	4	Korea Institute of Oriental Medicine	South Korea
3	Baumann, Stefan	3	University Medical Centre Mannheim	Germany
3	Choi, Ahnryul	3	Catholic Kwandong University	South Korea
3	Kandil, Emad	3	Tulane University School of Medicine	USA
3	Kang, Jeonghyun	3	Yonsei University College of Medicine	South Korea
3	Koutsouleris, Nikolaos	3	Ludwig-Maximilian-University	Germany
3	Liu, Xun	3	The Third Affiliated Hospital of Sun Yat-sen University	Peoples R China
3	Lo-Ciganic, Wei-Hsuan	3	University of Florida	USA
3	Maurice, Matthew J.	3	Cleveland Clinic	USA
3	Shiao, S. Pamela K.	3	Augusta University	USA
3	Yuvaraj, R.	3	University Malaysia Perlis	Malaysia

*Peoples R China, People's Republic of China; UK, United Kingdom; USA, United States of America*.

### Distribution by Topic

Table 4 shows the 10 most common WoS subject categories. Surgery ranked first, with 496 publications (15.9%), followed by Urology and Nephrology (*n* = 241; 7.7%), Medicine, General and Internal (*n* = 212; 6.8%), Neuroscience (*n* = 204; 6.6%), and Radiology, Nuclear Medicine and Medical Imaging (*n* = 172; 5.5%).

**Table 4:** The most productive Web of Science subject categories in gender-related articles in medical artificial intelligence.

**Rank**	**Web of Science subject category**	**Frequency**	**%**
1	Surgery	496	15.9
2	Urology and Nephrology	241	7.7
3	Medicine, General and Internal	212	6.8
4	Neurosciences	204	6.6
5	Radiology, Nuclear Medicine and Medical Imaging	172	5.5
6	Medical Informatics	168	5.4
7	Clinical Neurology	160	5.1
8	Multidisciplinary Sciences	156	5.0
8	Oncology	155	5.0
10	Engineering, Biomedical	145	4.7

**Figure 4:**
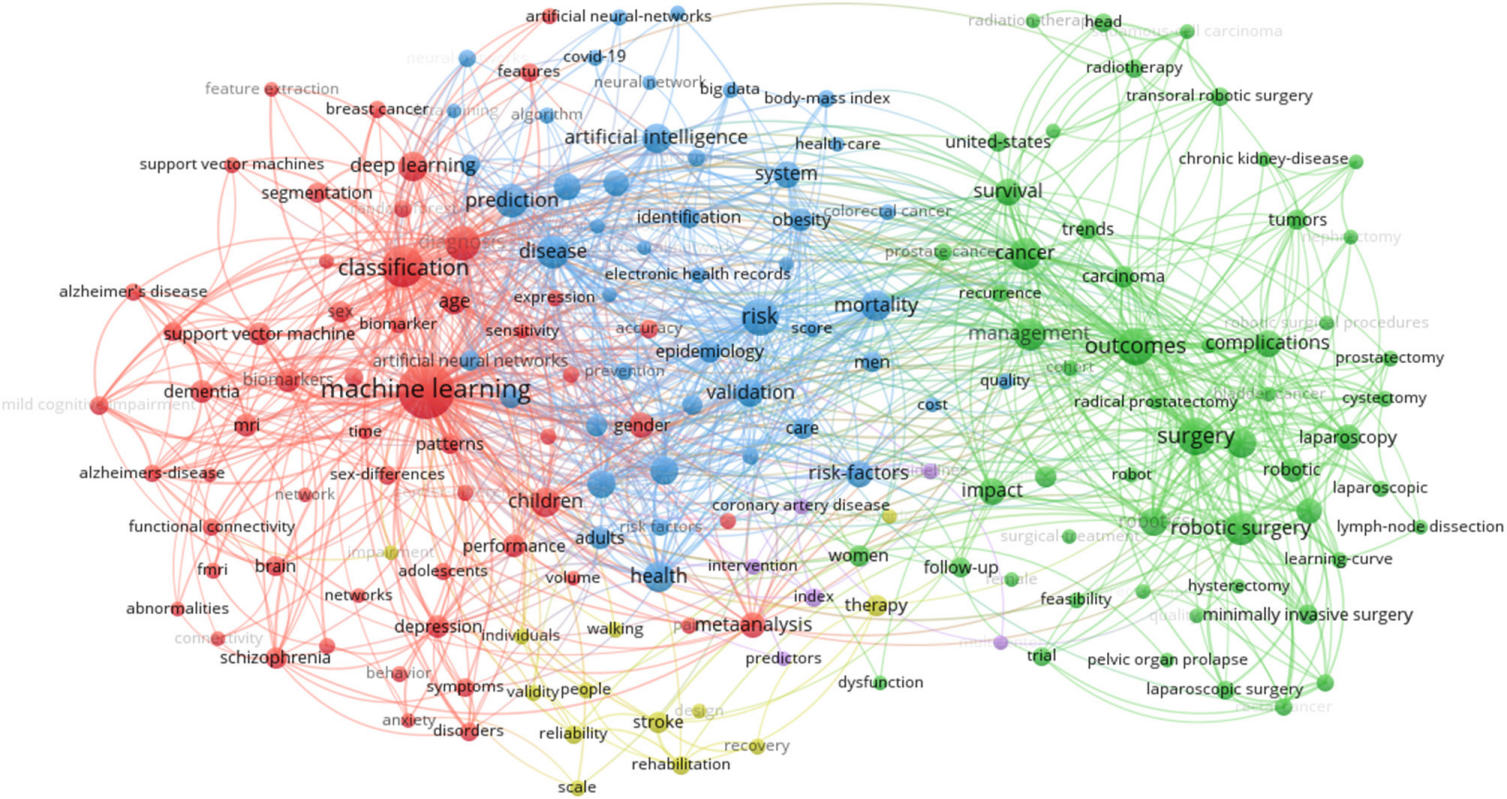
Network visualization map of keywords. Keywords included the author's keywords and keywords plus.

Figure 4 shows the network visualization map of keywords with a minimum occurrence of 20. Five clusters with 177 terms were obtained from the analysis: (i) a red cluster with 56 items focused on machine learning, classification, diagnosis, children, deep learning, and meta-analysis; (ii) a green cluster with 55 items focused on items focused on surgery, outcomes, robotic surgery, cancer, and management; (iii) a blue cluster with 47 items focused on risk, prediction, disease, mortality, health, artificial intelligence, and validation; (iv) a yellowish-green cluster with 13 items focused on stroke, therapy, rehabilitation, and reliability; and (v) a purple cluster with six items focused on index, guidelines, coronary artery disease, and intervention. Network visualization maps for keywords across the time periods were shown in Supplementary Figure 2.

### Distribution by Journal

The 3,110 papers were published in 1,281 journals. Table 5 lists the top 10 journals by the number of publications within the study period. The top 10 journals contributed 13.0% (403/3,110) of the total publications. *PLoS One* published the most articles on gender-related medical AI (*n* = 81; 2.6%), followed by *Surgical Endoscopy and Other Interventional Techniques* (*n* = 48; 1.5%), *Asian Journal of Surgery* (*n* = 44; 1.4%), and *Scientific Reports* (*n* = 43; 1.4%). Among the top 10 journals by publication number, *Surgical Endoscopy and Other Interventional Techniques* had the highest H-index (15), whereas the *Journal of Urology* had the largest number of citations per paper (55).

**Table 5:** The journals with the most gender-related articles in medical artificial intelligence.

**Rank**	**Journals**	**Number of papers**	**Number of citations**	**Citations per paper**	**H-index**	**Impact Factor (2020)**	**Web of Science subject category**
1	PLoS one	81	1,281	16.86	20	3.240	Multidisciplinary Sciences
2	Surgical Endoscopy and other Interventional Techniques	48	1,714	29.55	21	4.584	Surgery
3	Asian Journal of Surgery	44	242	5.5	9	2.767	Surgery
4	Scientific Reports	43	333	9	10	4.379	Multidisciplinary Sciences
5	JAMA Network Open	41	582	15.73	14	8.483	Medicine, General and Internal
5	Journal of Robotic Surgery	41	204	4.98	8	N/A	Surgery
7	Journal of Medical Internet Research	30	200	9.52	8	5.428	Health Care Sciences and Services; Medical Informatics
8	IEEE Access	28	190	6.79	6	3.367	Computer Science, Information Systems; Engineering, Electrical and Electronic; Telecommunications
9	Urology	24	374	16.26	10	2.649	Urology and Nephrology
10	Journal of Urology	23	1,320	55	16	7.450	Urology and Nephrology

*N/A: not available*.

### Characteristics of Top 9 Papers Most Frequently Cited

There were 44,711 citations in 3,110 publications. Table 6 shows the top 9 papers with the highest citation frequency. The top 9 papers accounted for 7.0 % (3,112/44,711) of the total citations and were cited 346 times, on average. The work of Wynants et al. (21) was the most cited paper (*n* = 567; 1.3%), followed by the study by Poplin et al. (22) (*n* = 382; 0.9%) and Aarts et al. (23) (*n* = 369; 0.8%). Among the top 9 papers, three were published in journals with an impact factor (IF) > 20, one in a journal with an IF between 10 and 20, three in journals with IFs between 5 and 10, and two in journals with an IF < 5.

**Table 6:** The papers with the most frequent citations of gender-related medical artificial intelligence.

**Rank**	**Title**	**First author**	**Journal**	**Impact factor (2020)**	**Year**	**Number of citations**	**Web of Science subject category**
1	Prediction models for diagnosis and prognosis of covid-19 infection: systematic review and critical appraisal	Wynants, Laure	BMJ-British Medical Journal	39.890	2020	567	Medicine, General and Internal
2	Prediction of cardiovascular risk factors from retinal fundus photographs via deep learning	Poplin, Ryan	Nature Biomedical Engineering	25.671	2018	382	Engineering, Biomedical
3	Surgical approach to hysterectomy for benign gynecological disease	Aarts, Johanna W. M.	Cochrane Database of Systematic Reviews	9.266	2015	369	Medicine, General and Internal
4	Robot assisted partial nephrectomy vs. laparoscopic partial nephrectomy for renal tumors: a multi-institutional analysis of perioperative outcomes	Benway, Brian M.	Journal of Urology	7.450	2009	365	Urology and Nephrology
5	Prospective randomized controlled trial of robotic vs. open radical cystectomy for bladder cancer: perioperative and pathologic results	Nix, Jeff	European Urology	20.096	2010	362	Urology and Nephrology
6	Alzheimer's disease diagnosis in individual subjects using structural MR images: validation studies	Vemuri, Prashanthi	Neuroimage	6.556	2008	298	Neuroimaging; Neurosciences; Radiology, Nuclear Medicine and Medical Imaging
7	Transoral robotic surgery: a multicenter study to assess feasibility, safety, and surgical margins	Weinstein, Gregory S.	Laryngoscope	3.325	2012	270	Medicine, Research and Experimental; Otorhinolaryngology
8	Compare: classification of morphological patterns using adaptive regional elements	Fan, Yong	IEEE Transactions on Medical Imaging	10.048	2007	254	Computer Science, Interdisciplinary Applications; Engineering, Biomedical; Engineering, Electrical and Electronic; Imaging Science and Photographic Technology; Radiology, Nuclear Medicine and Medical Imaging
9	Robot-assisted laparoscopic pancreatic surgery: single-surgeon experience	Giulianotti, Pier Cristoforo	Surgical Endoscopy and other Interventional Technique	4.584	2010	251	Surgery

## Discussion

Our bibliometric analysis of the gender-related articles in medical AI revealed major changes over the last 20 years. The number of publications and percentage of gender-related articles in medical AI fields continuously increased from 2001 to 2020, with a steep increase in the past 5 years. This change can be explained by both increased interest of AI and awareness of gender medicine. Due to the technological development including computing power and data storage, AI has been developed (24), leading to advances in researches and collaborative works in medical AI fields (15, 25). In addition, there have been continuous efforts to overcome this gender bias (4), although women used to be underrepresented in clinical research (26).

After the National Institute of Health (NIH) Revitalization Act of 1993 mandated the enrollment of women and ethnic minorities in clinical research in the USA (27), funding agencies such as the Canadian Institutes of Health Research (28), European Commission (29), and NIH (30) required consideration of sex and gender in study design, analysis, and reporting for grant applications. In addition, several editorial guidelines included gender-specific work [e.g., Animal Research: Reporting In Vivo Experiments (ARRIVE) (31), Sex and Gender Equity in Research (SAGER) (32), and International Committee of Medical Journal Editors (ICMJE) recommendations (33)].

Both the number of publications and RRI on gender-related medical AI have steadily increased for 20 years, showing the increase of research interests in related fields. The percentage of gender-related articles in medical AI doubled in the last 20 years to 6.5%, although this figure remains small. According to Sugimoto et al., in 2016, two-thirds of articles were gender-related reporting articles of clinical medicine and public health research, whereas one-third of such articles were for biomedical research (34). Geller et al. showed that 26% of NIH-funded randomized control trials in 2018 included sex as a covariate (35). Compared to other fields, medical AI had a low percentage of gender-related articles. This requires further study.

As the number of publications can only provide volumetric information, our analysis showed SI and CNCI across countries and over time. These two parameters can provide different perspectives on research trends (36). SI, the ratio of the percentage of publications related to the specific area in a given country to those worldwide, evaluates specialization. CNCI, which is the ratio of the observed to the expected number of citations in the same WoS category, shows the citation impact. For example, although Canada and Peoples R China had the highest percentage increase in the number of publications over the previous 20 years, Canada showed overspecialization and citation impact specifically in gender-related medicine AI research compared to the worldwide figures, whereas Peoples R China did not.

The USA had the most publications on gender-related medical AI between 2001 and 2020, with overall high CNCIs and SIs. As expected, the top 10 institutions and high-ranked authors were from the USA. According to the network visualization plot, most of the top 10 institutions were also well-connected through research networks. According to the bibliographic analysis of authors, it was possible to understand the relationships between authors. Author co-citation analysis visualized the intellectual structure of the scientific knowledge domain by calculating how often the author's work is cited with other authors (37), whereas bibliographic coupling showed the similarity relationships by calculating how often two papers are cited together (38). In addition, co-authorship analysis showed the cooperative and interactive relationships between authors, indicating the authors' willingness to write a paper together (39).

Surgery and Urology and Nephrology was the most common WoS subject category in our analysis. Similarly, *Surgical Endoscopy and Other Interventional Techniques, Asian Journal of Surgery, Urology*, and *Journal of Urology* were the journals that ranked high in the number of gender-related publications in medical AI. Surgery is one of the most developed areas in medical AI. AI can be applied pre-, intra-, and post-surgery, such as for preoperative risk prediction, imaging, 3D reconstruction, and robotic intervention (40, 41). As several studies reported the sex differences in prognosis after surgery (42–44), sex should be considered in AI surgery research. Urology is another area of interest in gender-related medical AI. There are anatomical, physiological, and pathophysiological urological differences between men and women (45). Hormones and metabolisms differ by sex, thereby affecting medical conditions (46). Furthermore, environmental and occupational exposures may differ by gender, which should be considered in gender medicine (47).

The network visualization map of keywords across the time periods showed that research topics have continuously expanded and changed over past two decades. In the first period (2001–2005), there was only two clusters; one was disease and the other was artificial neural networks and cancer. In the last period (2016–2020), there was 6 clusters including machine learning, risk, and surgery. These results can be used to guide future studies by listing the trending topics.

The citation analysis showed that gender-related medical AI had a high influence, with an average of 15 citations. The topics covered in the top 9 articles with the highest citations were surgery, imaging, and prediction models. The most cited article was the study of Wynants et al. (21), which systematically reviewed and critically evaluated all 232 predictive models for diagnosis and prognosis of COVID-19 including 169 studies. This study showed that gender is one of the frequent prognostic factors of COVID-19. As the COVID-19 pandemic has posed a threat to the global economic and health systems with high morbidity and mortality (48), COVID-19-related articles have recently dominated medical publishing in the last 2 years (49). The study of Poplin et al., the second most cited article, developed deep learning models using retinal fundus images to predict multiple cardiovascular risk factors including age and gender (22). The third most cited article was the article by Aarts et al. (23), which reviewed the effectiveness and safety of four types of hysterectomy surgeries in women with benign gynecological diseases. Interestingly, most of the top 9 articles were published in journals with an IF < 10. This demonstrates an increased interest in this field.

This bibliometric study has some limitations. First, like other bibliometric studies, the results can be affected by the search term and databases used. As we only used the WoS, we could not include publications in other electronic databases (e.g., PubMed or Embase). However, we selected the WoS covering a broad range of articles (50) and applied search strategies with high sensitivity. Second, there was a possibility of the inclusion of studies that had little to do with our topics. As we focused on showing macroscopic tendencies, studies were identified through search if they had AI-, medication-, and gender-related terms in titles, abstracts, or keywords, regardless of their topics. For example, the article by Roberts et al. (51), the originally identified as the second most cited article, suggested the structural topic models for surveys in political sciences; it was not presented in Table 6 for qualitative interpretation. Despite this, including gender-related words is meaningful because it covers gender in any way. Third, the number of citations can be biased by self-citations and time elapsed since publication. Lastly, non-English publications were not included.

To the best of our knowledge, this is the first bibliometric study to investigate the worldwide research output of gender-related medical AI by bibliometric analysis. This study concluded that gender-related research in medical AI increased over the past 20 years. Despite increased interest, gender-related research is still low in medical AI field and further research is needed.

## Data Availability Statement

The raw data supporting the conclusions of this article will be made available by the authors, without undue reservation.

## Author Contributions

JY and HG contributed to designing the study. HY contributed to acquisition and analysis of data. HY, JY, and HG contributed to interpretation of data. HY and HL contributed to drafting of the manuscript. JY and HG contributed to critical revision of the manuscript. All authors contributed to the conception and design and read and approved the final manuscript.

## Funding

The research leading to these results received funding from Ministry of Science and ICT under WISET202103GI01.

## Conflict of Interest

The authors declare that the research was conducted in the absence of any commercial or financial relationships that could be construed as a potential conflict of interest.

## Publisher's Note

All claims expressed in this article are solely those of the authors and do not necessarily represent those of their affiliated organizations, or those of the publisher, the editors and the reviewers. Any product that may be evaluated in this article, or claim that may be made by its manufacturer, is not guaranteed or endorsed by the publisher.
