# Supplementary material for: Global Research Trends of Gender-Related Artificial Intelligence in Medicine Between 2001–2020: A Bibliometric Study
Source: Front Med (Lausanne). 2022 May 17;9:868040. doi: 10.3389/fmed.2022.868040 (PMC9152019; doi:10.3389/fmed.2022.868040)
Supplement: Supplementary file 1 [file Table_1.DOCX]

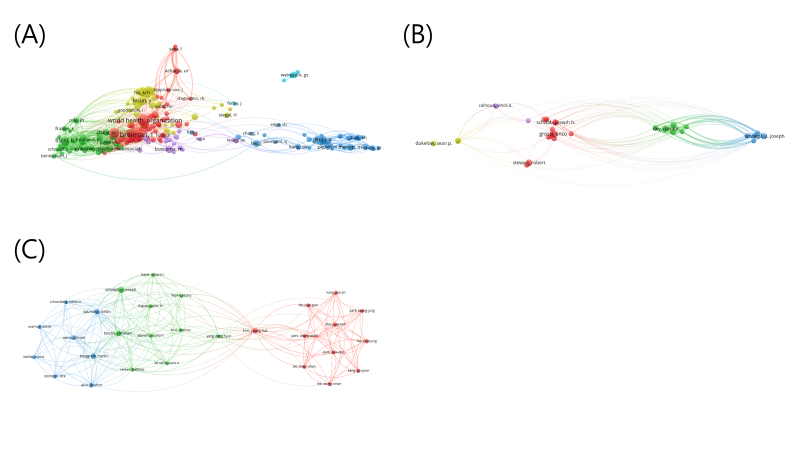


Supplementary Figure 1. (a) Network visualization map of co-citation of authors. (b) Network visualization map of bibliographic coupling of authors. (c) Network visualization map of co-authorship of authors.


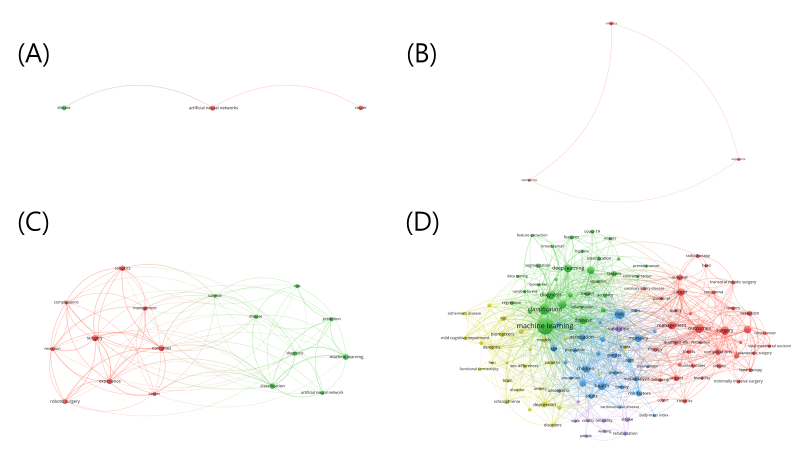


Supplementary Figure 2. (a) Network visualization map of keywords in 2001~2005. (b) Network visualization map of keywords in 2006~2010. (c) Network visualization map of keywords in 2011~2015. (d) Network visualization map of keywords in 2016~2020.
